# Comparative analysis of influenza epidemiological characteristics in Quzhou, China from 2016 to 2024

**DOI:** 10.1080/07853890.2025.2599618

**Published:** 2025-12-09

**Authors:** Hui Yang, Feipeng Chen, Bingdong Zhan, Shuangqing Wang, Zhao Yu, Zhidan Hua

**Affiliations:** a Department of Infectious Disease Control and Prevention, Quzhou Center for Disease Control and Prevention, Quzhou, Zhejiang Province, China; b Department of Infectious Disease Control and Prevention, Zhejiang Center for Disease Control and Prevention, Hangzhou, Zhejiang Province, China; c Department of Pulmonary and Critical Care Medicine, The Quzhou Affiliated Hospital of Wenzhou Medical University, Quzhou People’s Hospital, Quzhou, Zhejiang Province, China

**Keywords:** Influenza-like illness, COVID-19, epidemiological characteristics, virology, trend analysis

## Abstract

**Background:**

This study analyzed influenza-like illness (ILI) and influenza-positive rate (IPR) in Quzhou from 2016 to 2024 to assess the impact of the COVID-19 pandemic.

**Methods:**

The weekly surveillance data of ILI were collected from sentinel hospitals and network laboratories. Real-time reverse transcription-polymerase chain reaction (RT-PCR) was used to detect and identify the specific types of influenza viruses.

**Results:**

A total of 77,912 ILI cases were reported (ILI% = 3.9%). Both ILI% and IPR showed seasonal winter-spring peaks. A marked decline in ILI% (4.7% to 2.7%) and IPR (19.2% to 10.7%) was observed during the COVID-19 pandemic period (2020–2022), followed by a post-pandemic rebound. Children under 15 years old accounted for the highest proportion of ILI cases (65.9%). The 5–14 age group had the highest IPR (33.6%). Influenza A(H3N2) (39.2%) and B/Victoria (36.0%) were the predominant strains. The correlation between ILI% and IPR varied significantly across COVID-19 pandemic phases: weak positive pre-pandemic (*r_s_* = 0.325), moderate negative during the pandemic (*r_s_* = −0.597), and moderate positive post-pandemic (*r_s_* = 0.584). Age-stratified analysis revealed distinct correlation patterns in different COVID-19 pandemic phases, with the strongest correlation in the 15–59 age group (*r_s_* = 0.56, *p* = 0.004).

**Conclusion:**

The COVID-19 pandemic has significantly influenced influenza activity and altered the relationship between ILI% and IPR. The inconsistent correlation between ILI and IPR, especially during the COVID-19 pandemic, highlights the necessity of integrated virological surveillance and appropriate definition of ILI for effective influenza monitoring and public health response.

## 1. Introduction

Although influenza has been incorporated into infectious disease management worldwide, it continues to impose a significant disease burden and consume substantial healthcare resources annually, particularly affecting children, the elderly, and individuals with underlying medical conditions [1–3]. It is reported that up to approximately 1 billion people infected influenza globally each year, including 3–5 million severe cases and 290,000–650,000 deaths due to influenza-related respiratory diseases [4]. The all-cause excess mortality rate due to influenza in China was estimated to 6.9 to 17.2 per 100,000 population, resulting in approximately 96,000 to 240,000 all-cause excess deaths annually [5]. Surveillance of influenza-like illness (ILI) and etiological testing through sentinel hospitals constitute core components of the Global Influenza Surveillance and Response System (GISRS) and national surveillance systems. Key indicators include the proportion of ILI cases (ILI%) and the influenza positive rate (IPR), which reflect community transmission and laboratory confirmation, respectively [6]. It’s important to note that the definition of ILI is based on non-specific symptoms. The composition of ILI not only includes influenza cases but may also include similar symptom cases caused by other pathogens (such as respiratory syncytial virus, adenovirus, and SARS-CoV-2, etc.). Therefore, when conducting influenza surveillance using ILI, a comprehensive judgment should be made by combining IPR, as this can more specifically reflect the actual transmission level of influenza virus. The World Health Organization (WHO) has established standardized criteria for defining influenza-like illness (ILI), yet national definitions of ILI exhibit variations across different countries [7]. Additionally, different definitions of influenza-like illness have significant impacts on the monitoring results of influenza [8,9]. Even so, ILI monitoring remains a key indicator for early detection of influenza activity trends globally due to its cost-effectiveness and high sensitivity. China has established a nationwide influenza surveillance network, which has generated long-term, continuous, and comparable data, providing critical insights into the seasonality, dominant strain dynamics, and population distribution of influenza across different regions [10].

During the COVID-19 pandemic, non-pharmaceutical interventions (NPIs)-such as mask-wearing, social distancing, school closures, gathering restrictions, and limitations on cross-regional travel-significantly altered the ecology and seasonal circulation patterns of respiratory viruses in many parts of the world. This led to a notable reduction in influenza activity during 2020–2021 in multiple regions, followed by resurgences of varying degrees as interventions were relaxed and population susceptibility increased [11–13]. The dual transmission of SARS-CoV-2 and influenza virus will increase the difficulty of epidemic prevention and control as well as the challenges in response in the future. On the one hand, seasonal influenza will impose the pressure on the emergency medical support system, exacerbating the strain on limited medical resources. On the other hand, the monitoring of SARS-CoV-2 will disrupt the normal operation of influenza monitoring system to some extent, interfering with the analysis and prediction of influenza virus strains. Moreover, the predictive ability for potential new influenza virus strains causing a pandemic may be affected in short-term or even in the long-term [14].

Quzhou City is located in the west of Zhejiang Province (one of the southern provinces in China), with a total area of approximately 8,844.8 square kilometers and a permanent population of about 2.3 million. The population pyramid of Quzhou shows a narrowing at the bottom (children) and middle (young adults), and an expansion at the top (elderly), which is a typical population structure of deeply aging society. Before COVID-19 pandemic, the influenza in Quzhou has a clear peak in winter and spring, with occasional small peaks in summer, which is in line with the influenza epidemic characteristics of southern provinces in China. Vaccination and continuous monitoring, including surveillance of symptoms and pathogen are effective measures for influenza prevention and control. People aged 6 months and above without contraindications are recommended vaccinated against influenza, with priority given to medical staff, the elderly over 60 years, children over 6 months old, pregnant women, patients with chronic diseases and other high-risk groups. There are 15 comprehensive hospitals at or above the secondary level in Quzhou City. Two hospitals with strong capabilities, large outpatient volumes were selected as influenza monitoring sentinel hospitals, including one comprehensive hospital at the municipal level and one at the county level. Changes in healthcare-seeking behavior, adjustments in testing strategies, and viral interference and competition among different respiratory viruses during the COVID-19 period may have affected the relationship between ILI% and IPR. This, in turn, may have influenced the effectiveness of previously established syndromic surveillance indicators for early warning and intensity assessment. Based on surveillance data from sentinel hospitals and local network laboratories from 2016 to 2024, this study aims to explore the temporal distribution, seasonality, and interannual variation of ILI, ILI% and IPR in Quzhou, describe the temporal succession of dominant strains/subtypes, evaluate the correlation and time-lagged relationship between ILI/ILI% and IPR across different phases and age groups, and assess the impact of the COVID-19 pandemic on the association between these two indicators, so as to provide scientific basis for effective prevention and control of influenza in the post-COVID-19 pandemic.

## 2. Materials and methods

### 2.1. ILI surveillance

The surveillance data of ILI used in this study were obtained from Quzhou City Influence Surveillance Network, which was the part of the China Influenza Surveillance Information System (CISIS), from 2016 to 2024. The Quzhou City Influence Surveillance Network comprises two sentinel hospitals and one influenza network laboratory. Sentinel hospitals conduct ILI surveillance in all internal medicine outpatient departments, internal medicine emergency departments, fever clinics, and/or pediatric internal medicine outpatient departments, as well as pediatric internal medicine emergency departments. ILI is defined as a fever (body temperature ≥38 °C) accompanied by cough or sore throat according to the Chinese Influenza Surveillance Technical Guidelines (2017 edition) [15]. Medical staff in the surveillance clinics of sentinel hospitals record the number of ILI cases and the total number of outpatient and emergency visits by age group on a daily basis, according to the ILI case definition. The responsible department of each sentinel hospital collects and aggregates this data daily and enters it into the CISIS. Samples of ILI cases meeting the criteria (ILI cases that have not taken antiviral drugs within 3 days of onset) were collected, with a weekly target of 10–40 samples, aiming for an annual average of 20 samples per week, and sent to designated influenza network laboratory within two working days for etiological testing. The types of specimens collected for ILI cases include throat swabs (the main specimens), nasal swabs and nasopharyngeal swabs. The ILI% represents the proportion of ILI cases among the total outpatient and emergency department cases in the hospital [16].

### 2.2. Influenza virus detection and identification

Virus RNA extraction was completed by using RNeasy Mini kit (Qiagen, Dusseldorf, Germany), according to the reagent kit instruction. The influenza virus nucleic acid detection was carried out using real-time reverse transcription-polymerase chain reaction (RT-PCR) in accordance with the corresponding kit. The PCR reaction system and amplification conditions should be determined according to the selected kit, and cross-contamination should be avoided during the operation. Influenza virus identification performed using AgPath-ID Onestep RT-PCR kit (Applied Biosystems, Foster City, CA), with primers and the fluorogenic probes synthesized in accordance with the Chinese Influenza Surveillance Technical Guidelines (2017 edition) [15].

### 2.3. Quality control

Despite the significant changes in public health landscape and testing practices for respiratory viruses during the COVID-19 pandemic, our sentinel system adhered to the standardized case definition and a fixed weekly sampling range of 10 to 40 specimens, which is crucial for ensuring the comparability of data across different time periods. The sentinel hospitals and network laboratory strictly adhere to the requirements of national influenza surveillance program. Personnel involved in specimen collection, transportation, and laboratory testing received specialized training. The Quzhou Center for Disease Control and Prevention regularly conducts supervisory inspections of the influenza surveillance progress and ensures that any problems could be promptly corrected. The Zhejiang Provincial Center for Disease Control and Prevention annually evaluated the quality of influenza surveillance work every year.‌‌

### 2.4. Data analysis

Statistical analysis was performed using R software (version 4.2.1, R Foundation for Statistical Computing, Vienna, Austria). Chi-square test was used to compare ILI% across different years and periods of the COVID-19 pandemic. The positive rate of influenza virus nucleic acid detection in different years and age groups and the distribution of influenza virus subtypes across different age groups were also compared using Chi-square test. Spearman correlation analysis was used to examine the relationship between ILI/ILI% and IPR. Use the cross-correlation function (CCF) in the forecast package to conduct time cross-correlation analysis on ILI% and IPR. *p* < 0.05 was considered statistically significant. The different stages of the COVID-19 pandemic are announced in accordance with the progression of the epidemic within China, before the COVID-19 pandemic (before January 2020), during the pandemic (January 2020 to December of 2022), and after the pandemic (January of 2023 and after).

## 3. Results

### 3.1. Monitoring situation of ILI

From 2016 to 2024, the sentinel hospitals in Quzhou city reported a total of 77,912 ILI cases, with 1,997,262 outpatient and emergency cases, and the average ILI% was 3.9%. Among all the monitoring years, the highest ILI% was 4.8% in the year 2023, and the lowest was 1.8% in the year 2022. There was a statistically significant difference in ILI% among the years (*χ*^2^ = 5,245.231, *p* < 0.001) (Table 1).

**Table 1.** Age distribution of ILI cases in Quzhou, 2016–2024.

Surveillance years	Age(years)					Counts	OPD/ED	ILI%
	0–4 (%)	5–14(%)	15–24(%)	25–59(%)	≥60(%)			
2016	3459 (48.9)	1530 (21.6)	493 (7.0)	1127 (15.9)	471 (6.7)	7080	168235	4.2
2017	3532 (44.9)	1388 (17.6)	606 (7.7)	1700 (21.6)	644 (8.2)	7870	186438	4.2
2018	4614 (46.8)	1570 (15.9)	858 (8.7)	2224 (22.6)	590 (6.0)	9856	212980	4.6
2019	4835(42.9)	2139 (19.0)	1276 (11.3)	2487 (22.1)	533 (4.7)	11270	239658	4.7
2020	2868 (48.5)	682 (11.5)	556 (9.4)	1382 (23.4)	431 (7.3)	5919	145642	4.1
2021	1684 (36.4)	822 (17.8)	489 (10.6)	1029 (22.3)	600 (13.0)	4624	188615	2.5
2022	1256 (29.7)	1089 (25.7)	449 (10.6)	752 (17.8)	684 (16.2)	4230	234626	1.8
2023	5202 (35.8)	4639 (31.9)	1538 (10.6)	2375 (16.4)	775 (5.3)	14529	304487	4.8
2024	5538 (44.2)	4531 (36.2)	699 (5.6)	1532 (12.2)	234 (1.9)	12534	316581	4.0
Total	32988 (42.3)	18390 (23.6)	6964 (8.9)	14608 (18.8)	4962 (6.4)	77912	1997262	3.9

Abbreviations: OPD/ED, number of outpatients and emergency department visits.

**Figure 1.**
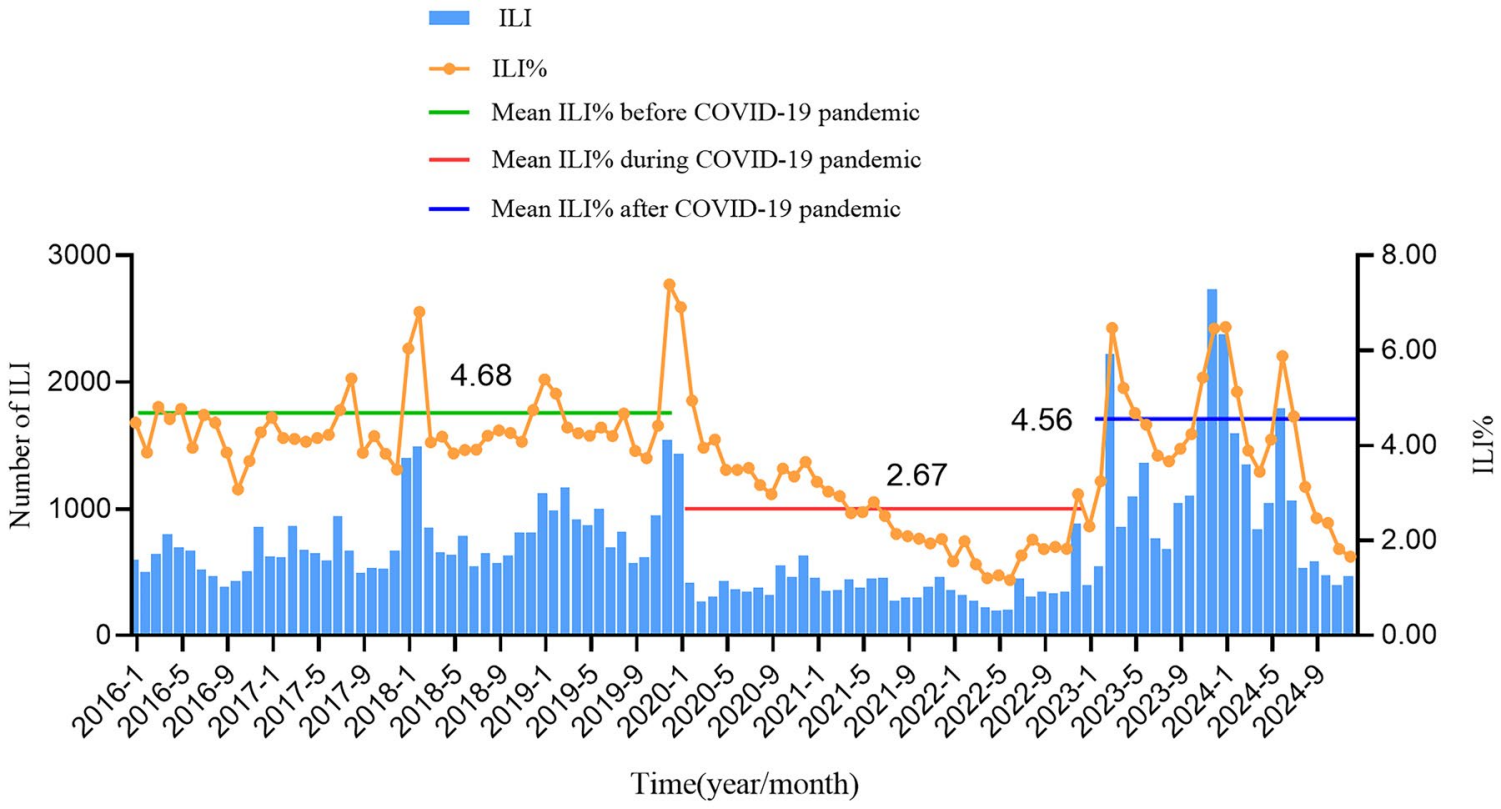
Time distribution of ILI and ILI% in Quzhou, 2016–2024.

The distribution of ILI and ILI% in Quzhou City from 2016 to 2024 shows a relatively obvious seasonality, mainly in winter and spring. The annual epidemic peaks are concentrated from December to March of the following year. There are small peaks in summer in some years (2016, 2017, 2022, and 2024), but the peak times are different. In 2017 and 2022, they both occurred in August, in 2016 in July, and in 2024 in June. The highest ILI% was 7.4% in December 2019, and the lowest was 1.8% in June 2022. The average ILI% was 4.7% before the COVID-19 pandemic, 2.7% during the pandemic, and 4.6% after the pandemic, showing a significant decrease during COVID-19 pandemic (*χ*^2^ = 3,620.232, *p* < 0.001), as shown in Figure 1.

Among the ILI cases reported by sentinel hospitals from 2016 to 2024, children under 15 years old accounted for the highest proportion at 65.9%, followed by the 25–59 age group at 18.8%, the 15–24 age group at 8.94%, and the ≥60 age group had the lowest proportion at 6.4% (Table 1).

### 3.2. Pathogenic detection

**Figure 2.**
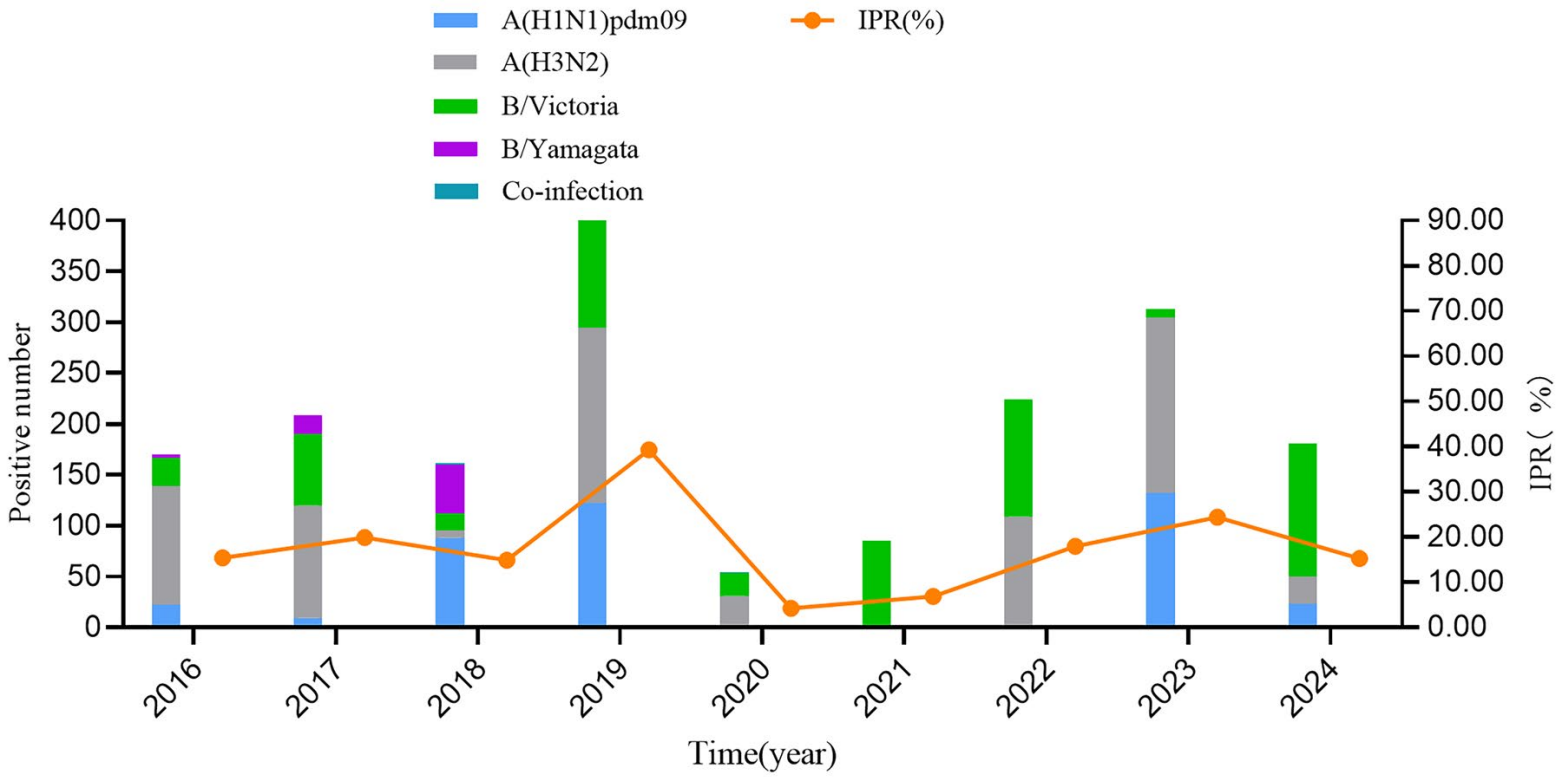
Etiological surveillance results of ILI samples in Quzhou, 2016–2024.

**Figure 3.**
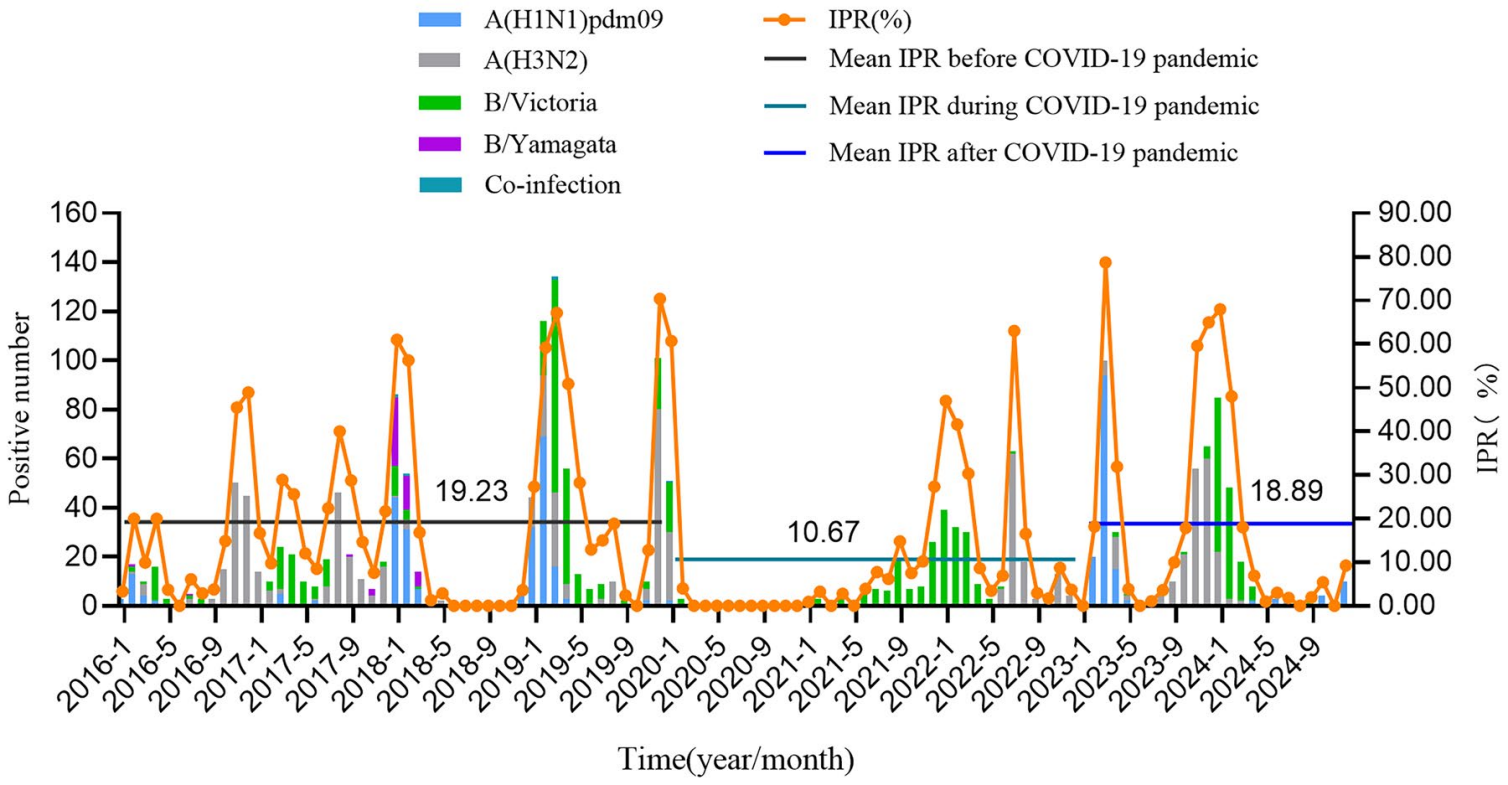
Trend of viral type and positive rate of ILI in Quzhou, 2016–2024.

From 2016 to 2024, the sentinel hospitals collected a total of 10,743 ILI samples, with 1,899 testing positive for influenza virus, resulting in a positive rate of 17.7%. The positive rate was the highest in 2019 at 39.4%, and the lowest in 2020 at 4.3%. There was a statistically significant difference in the IPR among different monitoring years (*χ*^2^ = 726.297, *p* < 0.001) (Figure 2). The IPR fluctuates significantly with the seasons, similar to the ILI%, mainly shows a peak in the winter and spring seasons. Some years (2016, 2017, 2019, 2021, and 2022) have a small peak in the summer, with the peaks occurred in July in 2016 and 2022, August in 2017 and 2019, and September in 2021. However, the years and months when the small summer peaks occurred are not exactly the same as those of ILI%. The average IPR (the proportion of the total number of positive samples in all weeks of a certain period to the total number of samples tested during the same period) was 19.2% before the COVID-19 pandemic (before January 2020), 10.7% during the pandemic (January 2020 to December of 2022), and 18.9% after the pandemic (January 2023 to December of 2024), showing a significant decrease during COVID-19 pandemic (*χ*^2^ = 264.488, *p* < 0.001), as shown in Figure 3.

From 2016 to 2024, the predominant influenza virus types in Quzhou City were: A (H3N2) (39.2%), followed by B/Victoria (36.0%), A (H1N1)pdm09 (20.2%), and B/Yamagata was the least, accounting for 3.7%. In addition, there were 4 samples showing co-infection, 2 samples were co-infected with both influenza A (H1N1) pdm09 and A (H3N2), 2 samples were co-infected with influenza A (H3N2) and B/Victoria, and no influenza B/Yamagata strain was detected after 2019 (Figure 2).

Except for the year 2020, each surveillance year exhibited distinct peaks of influenza virus detection. During the 2016–2017 winter-spring season, A(H3N2) was dominant, whereas the 2017–2018 was characterized by co-circulation of A(H1N1)pdm09, B/Yamagata, B/Victoria, and A(H3N2). In the 2018–2019 winter-spring period, A(H1N1)pdm09 and B/Victoria were predominant, with A(H3N2) also circulating; A(H3N2) re-surged in summer 2019. The 2019–2020 winter-spring season was dominated by A(H3N2) and B/Victoria, while in 2021–2022, B/Victoria was dominant, A(H3N2) prevailed in summer 2022. The following winter-spring period (2022–2023) was marked primarily by A(H1N1)pdm09, alongside A(H3N2). Finally, in the 2023–2024 season, A(H3N2) was initially prevalent, followed by the dominance of B/Victoria (Figure 3).

Among different age groups, the 5–14 age group had the highest positive rate (33.6%), while the ≥60 age group had the lowest (9.4%), with significant differences across all age groups (*χ*^2^ = 309.382, *p* < 0.001). No significant difference was observed in the positive rate between sex (*χ*^2^ = 3.510, *p* = 0.061) (Table 2).

**Table 2.** Pathological detection of ILI in different sex and age groups in Quzhou, 2016–2024.

Groups	Number of tests	Number of positive	Positive rate (%)	*χ*² value	*P* value
Sex				3.510	0.061
Male	4807	883	18.4		
Female	5936	1016	17.1		
Age groups				309.382	<0.001
0–4	921	129	14.0		
5–14	1319	443	33.6		
15–24	2102	368	17.5		
25–59	4974	825	16.6		
≥60	1427	134	9.4		
Total	10743	1899	17.7		

### 3.3. Relationship between ILI (or ILI%) and influenza positive rate (IPR)

**Figure 4.**
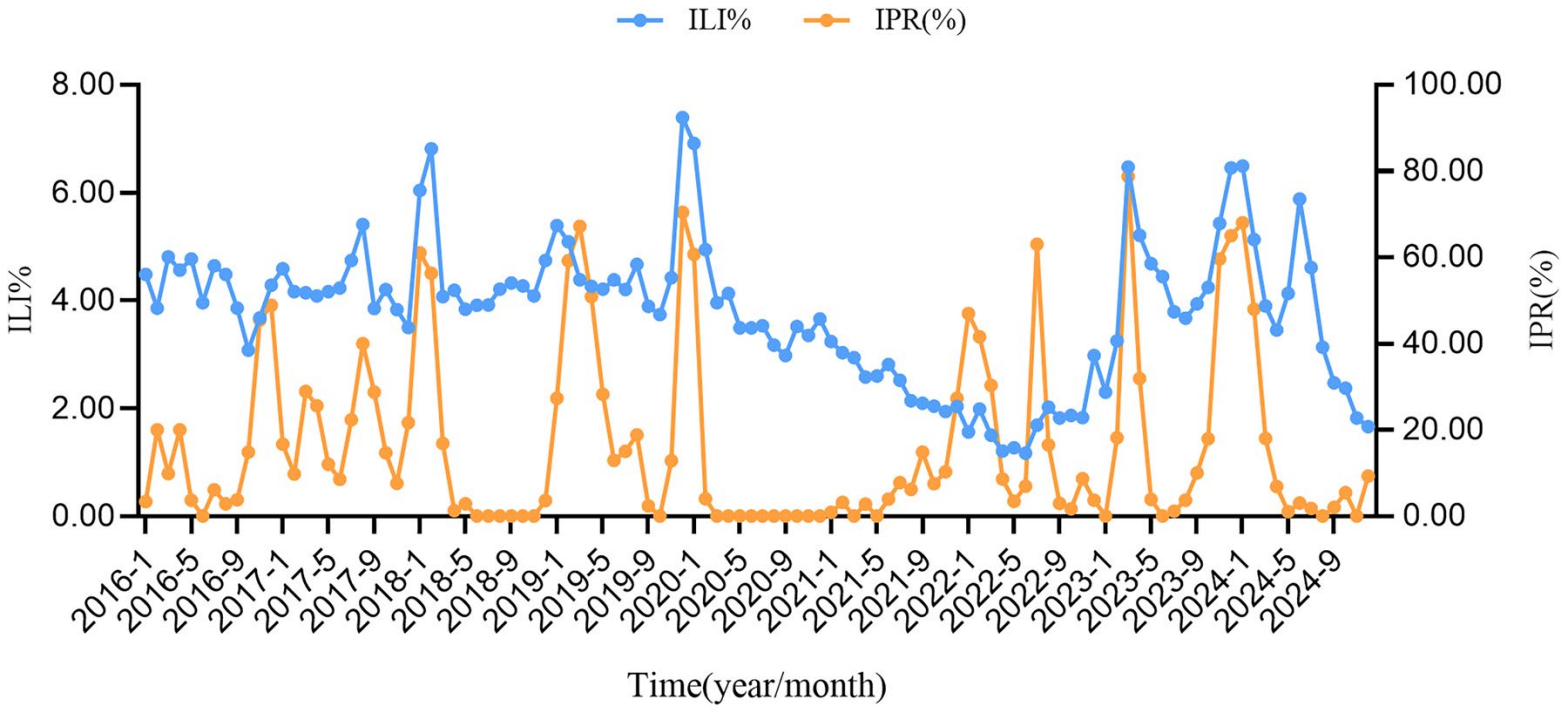
The correlation between ILI% and IPR in Quzhou from 2016 to 2024.

As shown in Figure 4, from the temporal distribution curves, it is evident that the trends of ILI% and IPR are not entirely consistent, particularly during 2020–2021. We assume that there is a monotonic positive correlation between ILI%/ILI and IPR. Correlation analysis indicated that, overall from 2016 to 2024, ILI% and IPR showed a weak positive correlation, with a correlation coefficient of 0.328 (*p* = 0.001). Time-lagged cross-correlation analysis revealed that the peak in ILI% occurred earlier than that of IPR, with a lead time of within one week (Table 3). To assess the impact of the COVID-19 pandemic on the relationship between ILI% and IPR, we compared their correlations across three phases: before, during, and after the pandemic. Before the pandemic, a weak correlation was observed between ILI% and IPR (*r_s_* = 0.325, *p* = 0.02). During the pandemic, a moderate negative correlation was found (*r_s_* = −0.597, *p* < 0.001). In the post-pandemic period, a moderate positive correlation was observed (*r_s_* = 0.584, *p* = 0.03).

**Table 3.** Result of time series cross-correlation analysis among ILI% and IPR in Quzhou, 2016–2024.

Monitoring Index	The correlation coefficient with ILI%								
	−4week	−3week	−2week	−1week	0 week	+1week	+2week	+3week	+4week
IPR(%)	0.12	0.18	0.29	0.32	0.33	0.38	0.34	0.28	0.19

Note: −1 week indicates a 1-week forward shift in the time distribution of ILI%, +1 week indicates a 1-week backward shift, and so on. *P* values are all less than 0.05.

**Figure 5.**
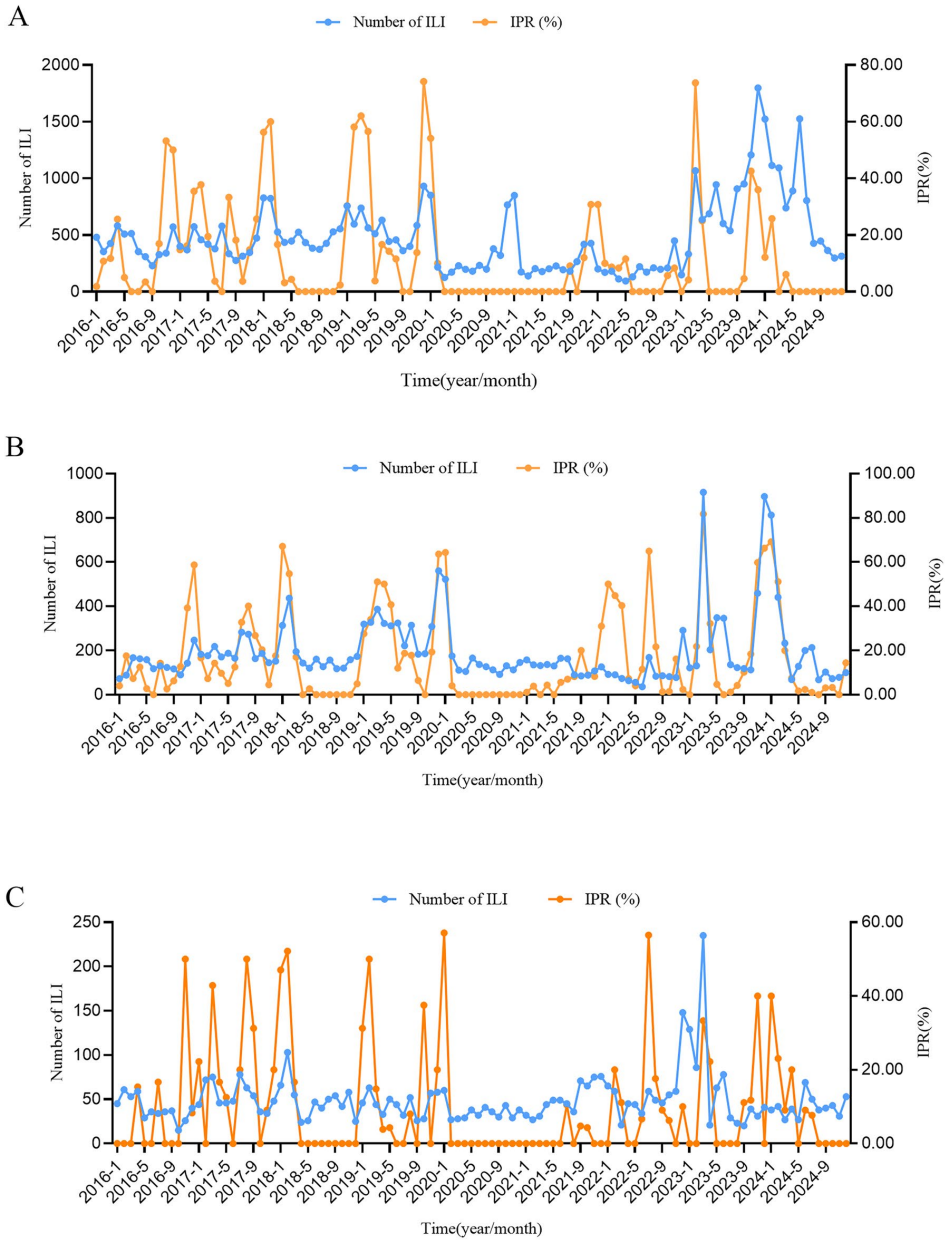
The correlation between ILI and IPR among different age groups in Quzhou from 2016 to 2024. A, the age group of 0–14; B, the age group of 15–59; C, the age group of ≥60 years.

To further investigate the influence of the COVID-19 pandemic on the correlation between ILI and IPR across different age groups, we stratified the analysis into three age categories: 0–14 years, 15–59 years, and ≥60 years. In the 0–14 years group, the correlation coefficients between ILI and IPR before, during, and after the pandemic were 0.393 (*p* = 0.006), 0.084 (*p* = 0.628), and 0.516 (*p* = 0.061) respectively; In the 15–59 years group, the correlation coefficients were 0.687 (*p* < 0.001), −0.237 (*p* = 0.164), and 0.56 (*p* = 0.004) across the three phases; In the ≥60 years group, the correlation coefficients were 0.377 (*p* = 0.008), 0.372 (*p* = 0.025), and 0.163 (*p* = 0.047) respectively (Figure 5).

## 4. Discussion

Based on sentinel surveillance data of Quzhou from 2016 to 2024, this study systematically described the evolving trend of influenza activity and its epidemiological and virological characteristics across the pre-pandemic, during-pandemic, and post-pandemic phase of COVID-19. During the COVID-19 pandemic, both ILI% and IPR decreased significantly, followed by a rebound in 2023–2024 to levels close to those observed before the pandemic, which are consistent with the overall trends reported in domestic and international studies [17,18]. On one hand, the notable decline during COVID-19 pandemic and the subsequent rebound support the strong suppressive effect of non-pharmaceutical interventions (NPIs) on influenza transmission and the accumulation of susceptible populations after their relaxation [13]. Measures such as mask-wearing, social distancing, and travel restrictions not only reduced viral exposure but may also have altered healthcare-seeking behavior, thereby depressing both ILI% and IPR. On the other hand, the rebound in 2023–2024 suggests that influenza activity can rapidly return to or even exceed pre-COVID-19 pandemic levels in the context of normalized behaviors, resumed domestic and international population mobility, and immunity gaps, highlighting the importance of strengthening prevention and vaccination strategies in the post-COVID-19 pandemic.

Influenza activity in Quzhou from 2016 to 2024 exhibited distinct seasonality, with occasional summer peaks in some years, consistent with patterns observed in other cities within the province and regions at similar latitudes [19–21]. However, the timing of summer peaks varied across years, which may be related to seasonal temperature and humidity in the eastern monsoon climate zone, school schedules, holiday travel, and the adaptability of dominant subtypes [22,23]. This underscores the need not only to maintain influenza control measures in winter and spring but also to sustain sufficient surveillance sensitivity and sampling during summer months to avoid underestimating unusual activity.

Children under 15 years accounted for the highest proportion of ILI cases, likely due to their relatively immature immune systems, less developed hygiene habits, and high-contact environments in school settings [24,25]. Additionally, parents may be more vigilant about children’s health, leading to higher care-seeking behavior. However, among children aged 0–4 years, although ILI accounted for the highest proportion, IPR was relatively low, suggesting a broader spectrum of respiratory pathogens causing influenza-like illness in this age group. Thus, promoting awareness of multiple respiratory infections and improving vaccine uptake for various preventable diseases are essential in pediatric public health. The lower IPR among the elderly may be associated with healthcare-seeking behavior, interference from non-specific respiratory symptoms due to chronic comorbidities, sampling bias, and vaccination coverage [26,27]. From 2016 to 2024, the predominant influenza subtypes in Quzhou were A(H3N2), B/Victoria, and A(H1N1)pdm09, with alternating dominance across years-a pattern generally consistent with national and regional trends [28–30]. It is worth noting that B/Yamagata was not detected locally after 2019, aligning with its global decline in circulation [31,32].

Influenza surveillance is conducted globally according to the ILI definition recommended by WHO. ILI is a non-specific indicator based on clinical symptoms, while IPR is a laboratory-confirmed specific indicator. Theoretically, the IPR should be positively correlated with the number of ILI cases. By analyzing the correlation between ILI and IPR, especially in different periods of COVID-19 pandemic, we can verify whether the clinical symptom and virological monitoring results in our influenza surveillance system are consistent. A high positive correlation can enhance our confidence in using ILI as an early warning signal for influenza activity. However, the temporal trends of ILI% and IPR were not fully consistent, showing only a weak correlation in our study. Time-lagged analysis indicated that ILI% peaked approximately one week earlier than IPR, reflecting the early warning capacity of syndromic surveillance while also possibly resulting from delays in specimen collection and laboratory reporting. When analyzed by phase, the correlation between ILI% and IPR varied significantly across different stages of the COVID-19 pandemic, suggesting that under conditions of strict NPIs and altered healthcare-seeking behavior, syndromic and laboratory indicators may become decoupled. Previous research showed that the number of ILI cases reported by the influenza surveillance system has no correlation with laboratory test results, the current definition of ILI has a relatively high negative predictive value but an unsatisfactory positive predictive value, but the monitoring of ILI plays a certain role in early warning and prediction of the epidemic during the influenza season [33]. While in our study, the stratified analysis by age groups reveals that the COVID-19 pandemic significantly changed the relationship between ILI and IPR across different populations. Prior to the pandemic, a consistent weak to moderate positive correlation was observed across all age groups, suggesting that ILI trends generally reflected true influenza activity. However, this relationship was markedly disrupted during and after the pandemic period in 0–14 age group, suggesting that the impact of COVID-19 pandemic on the relationship between ILI and IPR was age-dependent. Children and infants often have multiple respiratory pathogen infections, the current definition of ILI may not be suitable for influenza surveillance in children [16]. Another study suggested that the current definition of ILI is relatively strict for people aged 60 and above, but has a higher monitoring efficiency for those under 60 years [10]. While our study showed that, in the ≥60 years group, the overall correlation between ILI and IPR was weak but statistically significant.

The COVID-19 pandemic has altered the epidemiological patterns of respiratory pathogens such as rhinovirus, adenovirus, and respiratory syncytial virus (RSV) [34,35]. In the post-COVID-19 pandemic era, influenza prevention and control face multiple new challenges. It is imperative to strengthen integrated syndromic and etiological surveillance, optimize school-based sentinel surveillance and health education, and promote prioritized vaccination strategies for school-aged populations, coupled with pre-epidemic season publicity campaigns to enhance immunization coverage. Genetic and antigenic characterization of circulating strains should be enhanced to inform vaccine strain selection and immunization policy adjustments. The implementation of multiplex respiratory pathogen testing in outpatient, emergency, and community settings is also recommended to improve the accuracy of etiological diagnosis behind ILI% fluctuations and enhance the specificity of risk assessment.

However, our study has several limitations. First, since the number of outpatient and emergency visits by age groups were not available, we were unable to calculate age-specific ILI% and could only analyze the correlation between the absolute number of ILI cases and IPR over time. Second, due to the lack of influenza vaccination coverage data across years and age groups in Quzhou, we could not directly assess the impact of vaccination on the observed influenza epidemiology during the different periods of COVID-19 pandemic. Third, since no tests were conducted for other respiratory pathogens besides influenza, we were unable to assess the exact impact of SARS-CoV-2 on the prevalence of ILI cases. The sentinel surveillance protocol for ILI, including the case definition and the weekly sampling framework, remained consistent throughout the different periods of COVID-19 pandemic, thereby reducing the significant interference to the number of ILI cases or IPR caused by some external factors, such as the severity of the infection, public awareness, health-seeking behavior and testing intensity. However, we cannot completely rule out the potential impact of subtle differences in virus virulence, population immunity, or doctor compliance during different epidemic seasons or periods.

## 5. Conclusions

Influenza activity in Quzhou from 2016 to 2024 exhibited a seasonal pattern characterized by winter-spring peaks with occasional summer outbreaks. The COVID-19 pandemic significantly suppressed both ILI% and IPR, followed by a rapid rebound in the post-pandemic period. Influenza A(H3N2) and B/Victoria alternated in dominance, and no B/Yamagata strain was detected after 2019. Children and adolescents accounted for the majority of both clinical visits and confirmed infections. The relationship between ILI% and IPR was significantly disrupted during the COVID-19 pandemic and ILI% peaked approximately one week earlier than IPR. Enhancing integrated surveillance, age-tailored interventions, and optimized vaccination strategies in the post-COVID-19 pandemic era may improve the timeliness and precision of local influenza early warning and response. Our findings emphasize the importance of strengthening integrated virological surveillance alongside syndromic monitoring to accurately interpret epidemic dynamics, allocate resources efficiently, and develop targeted public health responses, especially for high-risk groups such as children and the elderly.

## Data Availability

The data are available from the corresponding author on reasonable request and with the permission of Quzhou Center for Disease Control and Prevention.
